# Tailoring Body Mass Index for Prediction of Obesity in Young Adults: A Multi-Centric Study on MBBS Students of Southeast India

**DOI:** 10.7759/cureus.12579

**Published:** 2021-01-08

**Authors:** Swikruti Behera, Alpana Mishra, Angeleena Esther, Ayaskant Sahoo

**Affiliations:** 1 Physiology, NRI Institute of Medical Sciences, Visakhapatnam, IND; 2 Community Medicine, Kalinga Institute of Medical Sciences, Bhubaneswar, IND; 3 Anaesthesia, NRI Institute of Medical Sciences, Visakhapatnam, IND

**Keywords:** obesity, young adults, body fat percentage, body mass index: bmi, roc curve, waist hip ratio, overweight

## Abstract

Introduction: Body mass index (BMI) has been used for a long period as a surrogative measure for obesity. But BMI does not differentiate between fat and nonfat tissue (blood, bone, and muscle) due to which it is not considered accurate anymore. But since BMI is easier to estimate and used widely for assessment of obesity, it is better if it is re-standardized according to the body fat percentage (BFP) of a specific population, community, and their ethnicity.

Objective: To estimate and propose the BMI cut-off values in young Indian population especially MBBS students taking BFP as a standard.

Design: This is a cross-sectional study. Anthropometric data (age, gender, height, weight, waist circumference, and hip circumference) were collected from the participants after taking consent. BMI was calculated using Quetelet’s Rule. BFP was estimated using Omron Body fat Monitor (HBF 385). It measures the BFP by the bioelectrical impedance (BI) method. Data were analyzed with appropriate statistical tests and receiver operating curve (ROC) curves were drawn to find the cut-off values of BMI to determine obesity.

Setting: The present study is a multi-centric study conducted in four medical colleges (two in each state; Odisha and Andhra Pradesh, India).

Participants: Apparently healthy MBBS students aged 18-24 years were included in this study. Students having any chronic or acute illnesses were excluded from the study. Out of 904 students contacted from four medical colleges, 863 (430 males and 433 females) consented and participated.

Results: Some 863 MBBS students have participated in this study. After adjusting for age, BMI was found to be higher in males. BMI was found to be 29.33 for males and in females it was 29.06. BFP was higher in females (34.23) as compared to males (20.77). Waist hip ratio was found to be higher in females (0.92) than in males (0.84). Whereas, fat free mass (FFM) and fat free mass index (FFMI) are higher in males, i.e., 56.24 and 18.48 respectively. Most appropriate cut-off value for obesity on ROC curve was found to be 22.09 (sensitivity 84.5%, specificity 83.46%) in males and that of females was 23.73 (sensitivity 85.26, specificity 81.23). Whereas, the conventional cut-off of 25 for males had sensitivity of only 46% and that of females was 70.5%. For total population BMI cut-off value was found to be 22.2 with 81% sensitivity and 74% specificity.

Conclusion: We propose the cut-off value for overweight/obesity in males to be 22.09 kg/m^2^ and for females to be 23.73 kg/m^2^ in young adult Indian population. These values were found to have more sensitivity and specificity than current BMI cut-off value.

## Introduction

Overweight and obesity are rapidly escalating problems in developing countries. But obesity has reached epidemic proportions worldwide and has more than doubled since 1980. According to the World Health Organization (WHO) report [1], in 2016, 1.9 billion adults 18 years and older were overweight, having a BMI greater than 25 kg/m2. Obesity is one of the important causes for increased incidence of noncommunicable diseases like diabetes, hypertension, etc. We have been using body mass index (BMI) as the identifying marker for obesity since long as it is more convenient and requires minimal equipment. But BMI does not discriminate between fat and fat free mass (muscle, blood, bone, and water). To estimate abdominal obesity, many studies have used anthropometric measurements such as waist circumference or waist-to-hip ratio (WHR) [2-3].

Overweight and obesity are defined as abnormal/excessive fat accumulation that presents a risk to health [4]. It is the accumulation of body fat that puts the person at risk for many serious medical conditions including heart disease, diabetes, and even certain forms of cancer. Measuring the fat percentage is the more accurate method for assessing the fatness and obesity rather than assessing it by using BMI which does not differentiate between fat and muscle [5-6]. Fat percentage limits are different for males and females as they have different physiology. Having said that, measuring the fat percentage accurately is not easily done and certainly not without equipment so it is always more practical to use BMI which is adjusted according to the population, community, and ethnicity.

The rigors of education are stressful for many students in the present scenario. Young students have to deal with high academic demands, social changes, living away from home, busy schedules, eating unhealthy food without parental monitoring, and not maintaining a proper exercise routine. This is reﬂected by weight gain and declining ﬁtness levels [7]. This not only has deleterious effects on physical well-being: stress-induced neglect of proper nutrition and lack of sufﬁcient physical activity but also can be detrimental to students’ mental health and social well-being in the long run.

 World is a diverse place with a lot of ethnic and cultural variations. Owing to diverse culture, eating habits and environmental condition, the body composition can vary from one country to another. Hence, BMI should be customized according to ethnicity and race.

The aim of this study is to analyze and standardize the BMI cut-off values in young Indian population especially MBBS students taking body fat percentage (BFP) into consideration.

## Materials and methods

This multi-centric study was carried out in four medical colleges situated in two states (Odisha, Andhra Pradesh) of India. Participants (both males and females) were enrolled in this study from four Medical Colleges of different cities in South and eastern part of India; namely, Berhampur, Bhubaneswar (Odisha), Vishakhapatnam, and Amalapuram (Andhra Pradesh). Sample size was calculated considering prevalence of obesity and overweight as 27% from previous study [8] and 4% allowable error (with design effect of 1.7). The sample was equally divided among four sites out of which 836 students have consented and participated in the study. Data were collected from June 2012 to June 2017 after obtaining due approval from Institutional Ethical Committee. This is a cross-sectional study which was conducted on young adults (aged between 18 and 24 years) studying MBBS in Medical Colleges.

The study subjects were explained about the purpose of the study and were assured about the confidentiality and anonymity of the information shared. Data were collected from the selected samples after written consent was obtained from respondents. Information regarding socio-demographic profile and anthropometric parameters were collected. 

Height was measured using a stadiometer (Prestige & IS IndoSurgical) to the nearest of 0.1 cm. The participant stood on the stadiometer without shoes with scapula, buttocks, and heels touching the vertical bar, the neck held in a natural nonstretched position, the heels were touching each other, and the head was held straight with the inferior orbital border in the same horizontal plane as the external auditory meatus (Frankfurt's plane).

Body weight and BFP were assessed using OMRON HBF-385 (Karada scan), Krell Precision (Yangzhou Co. Ltd., Yangzhou, PR China). Pre-standardization of the instrument was done. OMRON HBF-358-BW measures the BFP by the bioelectrical impedance (BI) method. Muscles, blood vessels, and bones are body tissues with a high-water content that conducts electricity easily. Body fat is a tissue that has little electrical conductivity. The HBF-358-BW sends an extremely weak electrical current of 50 kHz and less than 500 μA through your body to determine the amount of fat tissue. This weak electrical current is not felt while operating the HBF-358-BW. After entering the age, gender, and height of the subject in the settings, the participant was asked to stand barefooted on the unit with feet parallel and placed on the metal footplates. After the bodyweight is displayed, the handheld unit should be held firmly with finger wrapped around the plates in both palms and arms are extended at an angle of 90° to the body which should be kept straight at all times during measurement [9].

Waist and hip circumference were measured using a stretch-resistant measuring tape. Measurement for finding out hip circumference was taken from the maximum perimeter of the buttocks. The waist circumference was measured at the approximate midpoint between the lower margin of the last palpable rib and the top of the iliac crest [10]. All the measurements were made with the tape held snugly, but not too tight and the tape was held parallel to the floor.

Calculations

Waist-to-hip ratio (WHR) = waist circumference/hip circumference

BMI calculated using the formula -

 BMI = body weight (in kg) / [height (in metres)]2

 Fat free mass index (FFMI) calculated using the formula -

 FFMI = [body weight - (BFP × body weight)] / [height (in metres)]2 [11].

 Body fat percentage of 25%-35% in females and 13%-23% in males [4-5, 9] was considered normal. Overweight or obesity indicated by high body fat was defined as BFP >23% in males and >35% in females [9]. WHR ≥ 0.90 in males and ≥ 0.85 in females is considered abnormal as per WHO guidelines [10].

Students having fever or any other form of acute or chronic illness at the time of study were excluded from the study.

Analysis and reporting

The data were entered into MS Excel and analysis was done using the SPSS 21 software. Mean, standard deviation, student’s t test, and chi square test were done for generation of results. Receiver operating curve (ROC) was plotted using the data for predicting cut-off values using BFP as a standard for predicting obesity and overweight.

## Results

Out of 904 students invited for study, 863 students (430 males and 433 females) have consented and participated in the study.

Body fat percentage is higher in females (34.23) as compared to males (20.77). Whereas, FFM and FFMI are higher in males, i.e., 56.24 and 18.48 respectively.

Table 1 shows that there was no difference in the BMI of males (23.75) and females (23.69). The BFP of both genders was found significant (i.e., 22.09% in males versus 31.06% in females) [8]. FFMI (in males 18.22 and 16.13 in females) and WHR (0.87 in males and 0.82 females) were also different and significant in both genders. Abdominal obesity was higher in females (30%) as compared to males (26%) but was not statistically significant.

**Table 1:** Anthropometric profile and characteristics of study participants (n=863). SD, standard deviation; BFP, body fat percentage; BMI, body mass index; FFMI, fat free mass index; WHR, waist-to-hip ratio p value <0.05 is significant

Characteristics	Males mean (SD)	Females mean (SD)	p value
Age (in years)	19.29(1.25)	18.63(1.09)	0.0001
Weight (in kilograms)	66.60(13.03)	57.81(11.43)	0.0001
Height (in meters)	1.68(0.07)	1.56(0.06)	0.0001
BMI	23.75(4.04)	23.69(4.40)	0.575
Waist circumference (in cm)	59.96(26.43)	61.58(22.13)	0.328
Hip circumference (in cm)	68.89(29.55)	75.26(27.19)	0.01
WHR	0.87(0.05)	0.82(0.06)	0.0001
BFP	22.09(5.80)	31.06(5.62)	0.0001
FFMI	18.22(2.35)	16.13(1.89)	0.0001

Table 2 depicts the age adjusted mean of selected characteristics. After adjusting for age BMI was higher in males (29.33) than in females (29.06). WHR was found to be higher in females (0.92) than in males (0.84).

**Table 2:** Gender wise distribution of age adjusted mean for selected characteristics of the study population. BMI, body mass index; WHR, waist-to-hip ratio; BFP, body fat percentage; FFMI, fat free mass index; SE, standard error p<0.05 is significant

Characteristics	Male mean (SE)	Female mean (SE)	p value
BMI	29.33(3.02)	29.06(3.65)	0.0001
WHR	0.92(0.04)	0.84(0.05)	0.0001
Waist circumference (in cm)	88.94(6.47)	80.46(8.01)	0.0001
BFP	20.77(4.35)	34.23(4.67)	0.0001
Fat free mass	67.40(6.05)	56.24(4.47)	0.0001
FFMI	22.49(1.75)	18.48(1.57)	0.0001

According to Table 3, distribution of BMI categories among males and females was not statistically significant whereas BFP was differentially distributed among males and females and was significant. WHR was found to be statistically significant too.

**Table 3:** Association of anthropometric parameters with gender. BMI, body mass index; WHR, waist-to-hip ratio; BFP, body fat percentage; BFP >23% in males and >35% in females were taken overfat/high [9]. Waist circumference >90 cm in males and > 80 cm in females is considered high. WHR >= 0.90 in males and >= 0.85 was considered high [10]. p <0.05 is significant

Characteristics	Male	Female	
BMI levels	N (%)	N (%)	
<18.49	40(9.30)	40(9.24)	Chi Sq= 7.396 p value=0.06
18.5-24.99	247(57.44)	266(61.43)	
25-30	117(27.21)	88(20.32)	
>30	26(6.05)	39(9.01)	
Waist circumference			
<80 cm	400(93.02)	306(70.67)	Chi Sq= 74.014 p value=0.0001
80-90 cm	22(5.12)	73(16.86)	
>90 cm	8(1.86)	54(12.47)	
WHR			
Normal	318(73.95)	299(69.05)	Chi Sq= 2.5422 p value=0.111
High	112(26.05)	134(30.95)	
BFP			
Normal	133(30.93)	277(63.97)	Chi Sq= 94.45 p value=0.0001
High	297(69.07)	156(36.03)	
BMI			
Normal	287(66.74)	306(70.67)	Chi Sq= 1.546 p value=0.214
High	143(33.26)	127(29.33)	

**Figure 1:**
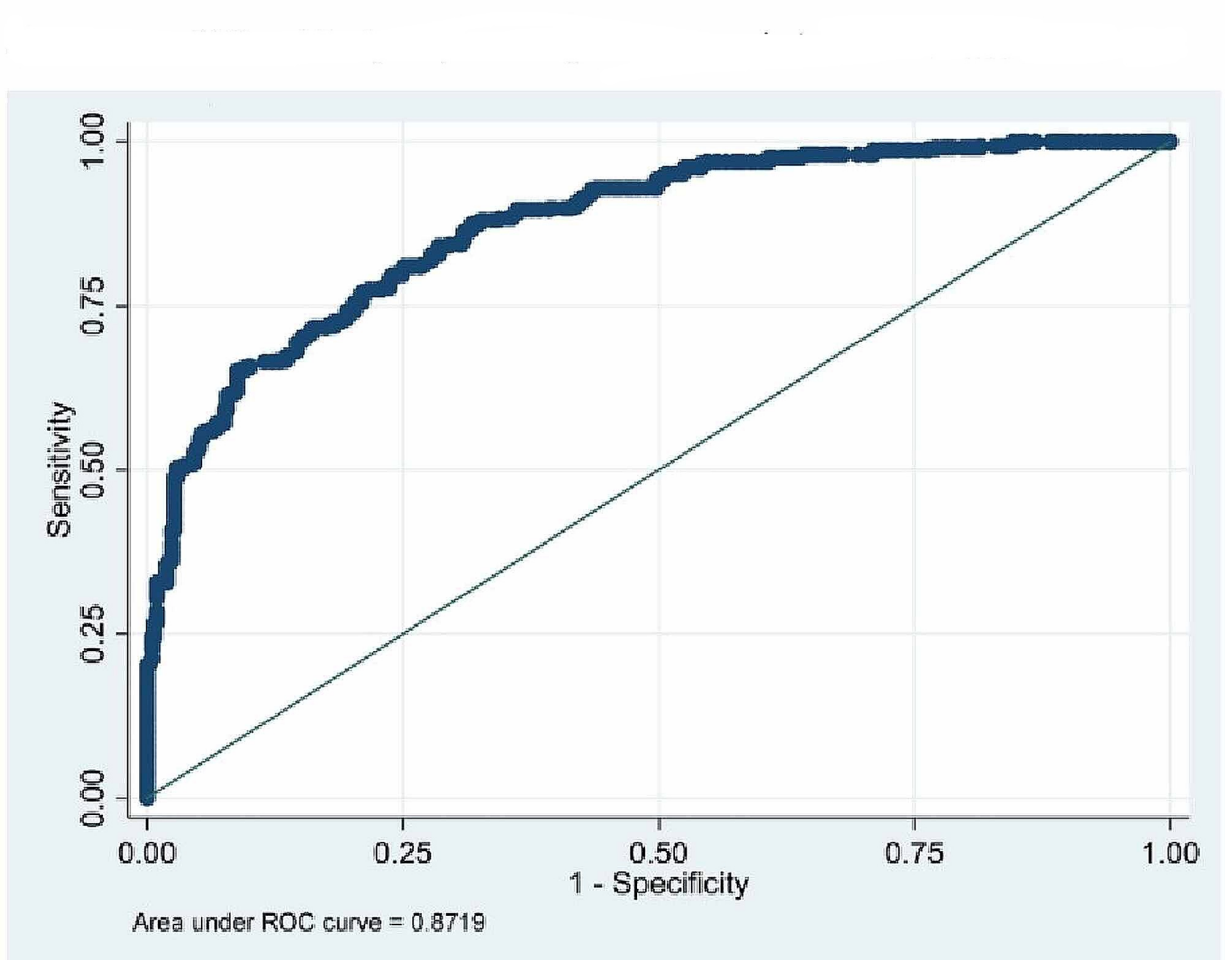
ROC on BMI and BFP (both males and females). ROC, receiver operating curve; BMI, body mass index; BFP, body fat percentage

**Figure 2:**
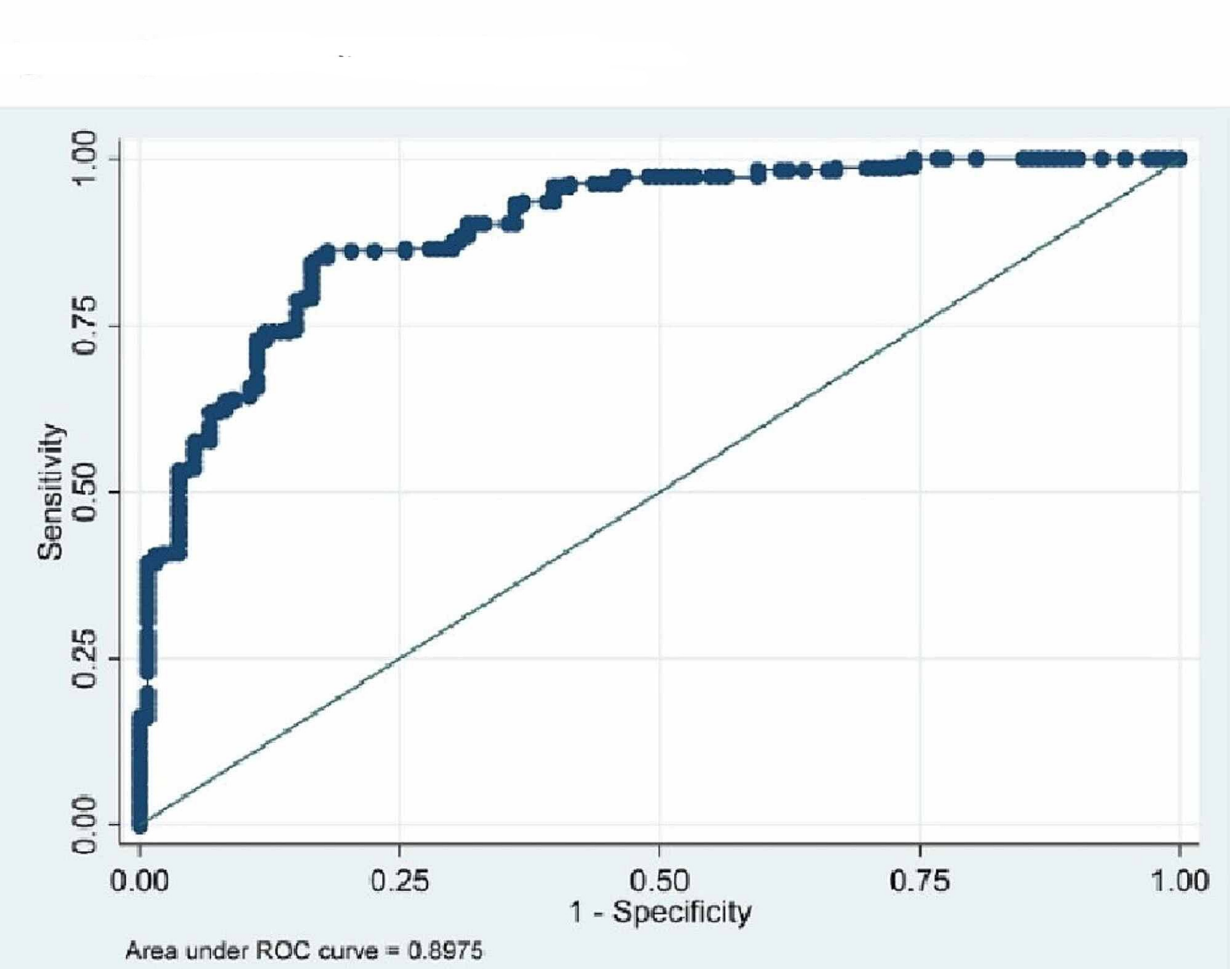
ROC curve on males (BMI vs. BFP). BMI, body mass index; BFP, body fat percentage; ROC, receiver operating curve

**Figure 3:**
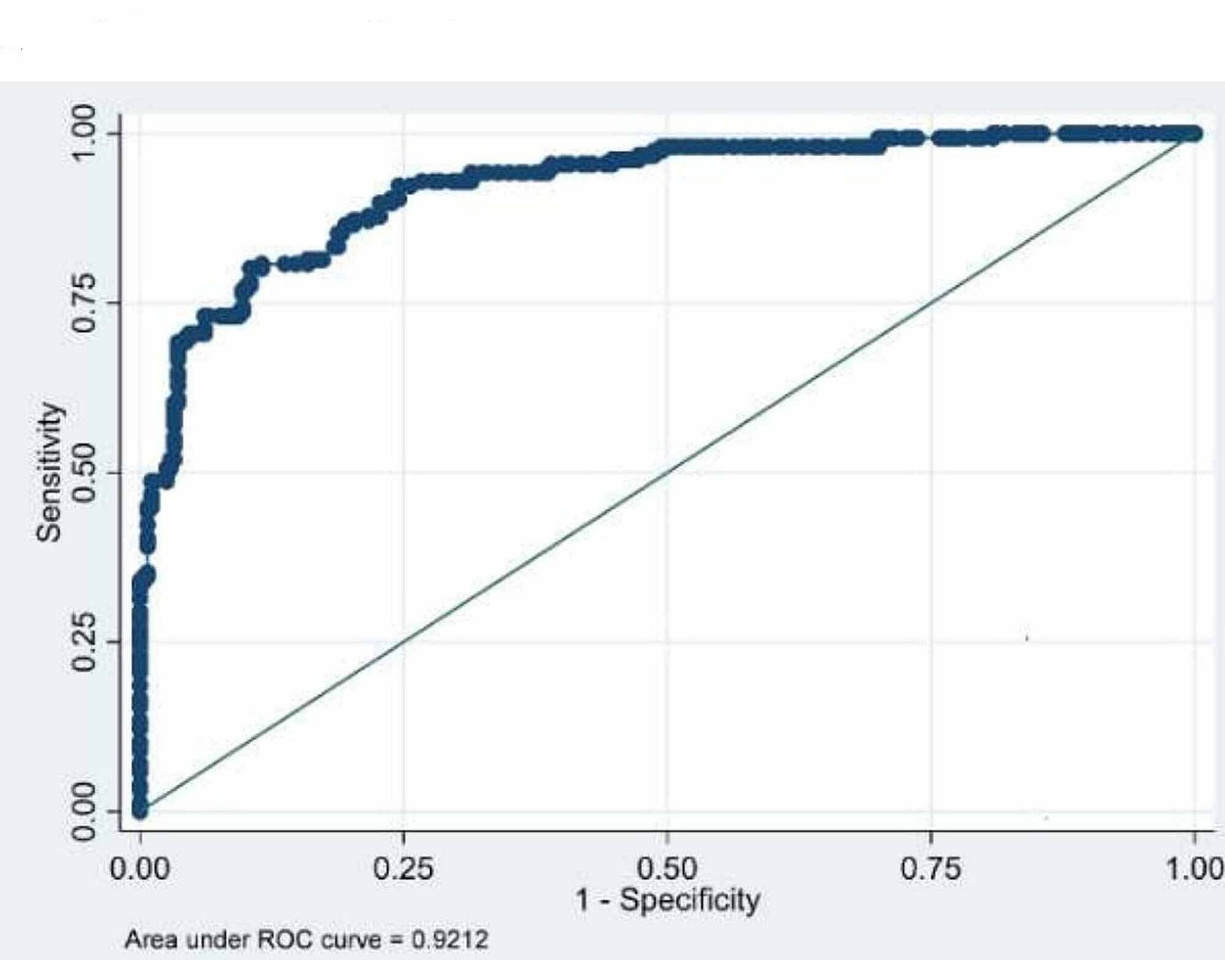
ROC curve on females (BMI vs. BFP). BMI, body mass index; BFP, body fat percentage; ROC, receiver operating curve

The ROC curves were drawn to find out appropriate cut-off value of BMI to determine obesity (Tables 3-4 and Figures 1-3). The cut-off value for obesity in males was found to be 22.09 with 84.5% sensitivity and 83.46% specificity. The BMI value predicting obesity in females was found to be 23.73 with sensitivity and specificity 85.26 and 81.23 respectively. Whereas, the conventional cut-off of 25 for males had a sensitivity of only 46% and that of females was 70.5%. For total population BMI cut-off value was 22.2 with 81% sensitivity and 74% specificity.

**Table 4:** Identification of BMI threshold and diagnostic assessment of obesity, considering BFP as gold standard. BMI, body mass index; BFP, body fat percentage; AUROC, area under receiver operating curve

Study population	BMI cut-off value	AUROC	Sensitivity	Specificity
Male (n=430)	22.09	0.8975	84.51	83.46
Female (n=433)	23.73	0.9212	85.26	81.23
Total (n=863)	22.2	0.8719	81.02	74.88

## Discussion

Body mass index has been in use since mid-19th century. BMI has been a useful and convenient tool owing to its universal acceptance, feasibility, and convenience. BMI has been used for long as the assessor for fitness and fatness but BMI has the disadvantage of not distinguishing between fat and muscle as it has been proved over the time by many [12-13]. It is fat content which increases the risk of several diseases (noncommunicable and communicable). Use of BFP and FFM as the marker of obesity is more appropriate as proved by various researchers all over the world. But owing to the fact that BMI is the easiest and most widespread method to assess obesity, it is still used widely all over world as the proxy measure for health and fitness.

The WHO expert committee meeting [14] had proposed BMI cut-off points 18-24.9 kg/m2 for normal, 25.0-29.9 kg/m2 for overweight, and >30.0 kg/m2 for obese. Taking the fact into consideration that though Asians in general have lower BMI, health risks related to obesity are also occurring at lower BMI; the Regional Office for Western Pacific Region of WHO, the International Association for study of Obesity and the Obesity Task force had proposed a separate classification for Obesity in Asia in 2000. This led to the proposal that adult overweight was specified in Asians as BMI over 23.0, and that obesity was specified over 25.0 (WPRO criteria) [15]. Aging is usually accompanied with a progressive increase in ratio of fat to lean mass owing to several contributing factors. But in present times stressful academic and professional studies have led to erratic lifestyle, lack of physical activity which in turn may also increase the total BFP [16]. In our study we found males and females to have significant difference among all anthropometric parameters except BMI and waist circumference (Table 1) but age adjusted mean found every parameter to be significant (Table 2). Erdembileg et al. [17] found the relation between BMI and body fat deposit or parameters constituting metabolic syndrome to be gender and age specific for Japanese workers.

Incidence of noncommunicable diseases in developing countries like India is on rise [18] more so at a young age [19]. The foundation of good health starts from a young age and the criteria to assess the health should be customized according to the need of the population. Aziz et al. [20] and Lim et al. [21] found that on following the revised guidelines the prevalence of obesity increased in pregnant women and in chronic obstructive pulmonary disease (COPD) patients respectively which will help in planning the intervention. As found in our study BFP and WHR were found more significant than the current BMI guidelines (Table 3). Fat percentage should be the ideal reference point for assessment of fatness.

From the ROCs (Figures 1-2 and Table 4) we found the cut-off value of BMI as 22.04 kg/m2 for males (with 84.5% sensitivity and 83.46% specificity) and 23.73 kg/m2 for females (85.26 and 81.23 specificity and sensitivity respectively) with highest sensitivity and specificity. Singh et al. [22] have proposed a higher cut-off value of 23.85 kg/m2 for males using ROC curve. Similarly, Dudeja et al. [8] in 123 North Indians (86 males and 37 females) have proposed a BMI cut-off value of 21.5 kg/m2 corresponding to 25% body fat for males and 19 kg/m2 for females. Fat percentage varies in males and females and so does the WHR [5, 7]. So, keeping one reference value for both males and females is unfair and inaccurate to assess the overweight and obesity.

We have used BI as the method to assess the BFP in the participants which is a fast, practical, noninvasive, and widely used method. Several studies have validated the use of BI as a standard for body fat estimation [23-24]. BMI is specific for race and hence, we agree to the conclusion of researchers like Piers et al. [25], who have concluded that BMI is useful and convenient for population studies but ill-applied in individuals. The need for population, ethnicity, and gender specific BMI cut-off is imperative and should be used with more precision especially in Indian population where the culture and food habits vary compared to other countries in Asian subcontinent.

Our study has included only young adults pursuing MBBS studies. All the data collected were from the southeastern part of India which might or might not be a representation of whole India. BMI cut-off value might change according to age. So, there is need to collect more data from older age groups as well as from other regions of the country (north and western regions of India). 

## Conclusions

We propose the cut-off value for overweight/obesity in males to be 22.09 kg/m2 and that for females to be 23.73 kg/m2 in young adult Indian population which have more sensitivity and specificity than current BMI cut-off value. Using these values for defining obesity in Indian population can help healthcare providers to educate, prevent, and treat various diseases where obesity might play an important role.
